# *Staphylococcus* biofilm dynamics and antibiotic resistance: insights into biofilm stages, zeta potential dynamics, and antibiotic susceptibility

**DOI:** 10.1128/spectrum.02915-24

**Published:** 2025-03-26

**Authors:** T. Lavoie, K. E. Daffinee, M. L. Vicent, K. L. LaPlante

**Affiliations:** 1 College of Pharmacy, University of Rhode Island, Kingston, Rhode Island, USA; 2 Providence Veterans Affairs Medical Center, Providence, Rhode Island, USA; 3 Division of Infectious Diseases, Warren Alpert Medical School of Brown University, Providence, Rhode Island, USA; Innovations Therapeutiques et Resistances (INTHERES), Toulouse, France

**Keywords:** biofilm, *Staphylococcus*, stages, *Staphylococcus aureus*

## Abstract

*Staphylococcus* spp. infections often involve biofilms, but standard antibiotic minimum inhibitory concentration (MIC) testing used to determine treatment evaluates planktonic bacterial growth only and does not account for biofilm presence, strength, or growth stage. To aid in determining a cost-effective method to solve this issue, we built upon *in vitro* methods initially published by Stepanovic et al. used to determine weak and strong biofilm formations. First, we determined 115 unique *S. aureus* isolate biofilms at 2, 4, 6, 8, 16, and 24 h to classify the hourly stages of biofilm development based on statistically significant final growth results (*P* < 0.001): stages one (0–6 h), two (6–16 h), three (16–24 h), and four (>24 h). Next, to further evaluate *in vitro* biofilm strength, electrostatic differences were measured through zeta (ζ)-potential for strong and weak biofilm producers at early and late stage-formed biofilms. The early stages of weak biofilm formers had a greater negative electrostatic charge when compared to strong biofilm formers. Meanwhile, strong biofilm formers began early stages with less negative charges before increasing the negative electrostatic charge by stage-four biofilm. At all time points, weak biofilm-forming isolate mean ζ-potentials were significantly more negative than strong biofilm formers (*P* = ≤0.04). Finally, to elucidate minimum eradication concentrations for biofilms, we treated stage-four biofilms with progressively higher concentrations of either daptomycin, vancomycin, or levofloxacin. Daptomycin was the only antibiotic to achieve ≥75% reduction in biofilm viability, seen at 32–256 μg/mL (64–512× MIC), and significantly reduced residual biofilm across all strong and weak biofilms. Biofilm findings showed an unexpected initial biofilm decrease in response to lower concentrations of antibiotics, followed by an increase in biofilm biomass at higher antibiotic concentrations. While higher antibiotic concentrations can be used to overcome bacterial resistance and eliminate infections, our results suggest that antimicrobial resistance is observed, regardless of bacterial biofilm strength, and that there may be an optimal treatment concentration window for achieving maximum kill. Our data add to the increasing evidence of biofilms’ role in recurrent infections and the importance of antibiotic concentration.

**IMPORTANCE:**

This work is significant, as it addresses a critical gap in standard antibiotic testing by focusing on the unique characteristics of biofilm-forming *Staphylococcus aureus* infections, which are major contributors to recurrent and chronic infections. Unlike traditional MIC testing that evaluates planktonic bacteria, this study emphasizes the importance of biofilm presence, growth stages, and electrostatic properties in determining treatment strategies. By classifying biofilm development into distinct stages in an easily reproducible assay and measuring the biofilm zeta-potential for key differences and overall biofilm response to multiple standard antibiotic concentrations, this research provides valuable insights for the future of biofilm *in vitro* work. Furthermore, it highlights the efficacy of daptomycin in eradicating biofilm while identifying possibilities of optimal antibiotic concentration windows, a critical consideration for mitigating resistance and achieving effective infection control. These findings underscore the necessity of tailoring treatment to biofilm-specific dynamics, offering a path toward more effective therapeutic approaches for biofilm-associated infections.

## INTRODUCTION

Biofilms increase the pathogenicity of bacteria, often causing serious infections, and account for up to 80% of all chronic and recurrent infections (1–4). The most predominant biofilm-forming pathogen, methicillin-resistant *Staphylococcus aureus* (MRSA), is listed as a serious threat by the Centers for Disease Control and Prevention (5, 6). This ability to form antibiotic resistant biofilms composed of an extracellular polymeric substance (EPS), a complex composition of extracellular DNA (eDNA), proteins, and polysaccharides, makes microbial eradication difficult (5, 7–9). The development of the MRSA biofilm is often discussed in stages but has not been evidently defined in a manner, which can be easily studied with common *in vitro* methods to test antimicrobials against various biofilm types. To better ensure optimal *in vitro* therapy for infections involving biofilm-producing organisms, the synthesis and proliferation of the matrices should be clearly defined. Utilizing these new definitions, antibiotic development can target specific stages of biofilm growth and have a greater clinical impact on patient outcomes.

Biofilm development of all organisms reportedly occurs in at least four stages: (i) attachment, (ii) accumulation, (iii) maturation, and (iv) dispersal (3, 10). The first stage of biofilm growth includes reversible and irreversible attachments involving bacterial adhesion within or on surfaces of the host. While it is a result of several factors, the attachment process importantly involves electrostatic charge-mediated bonding. Most bacteria initially exhibit a negative ζ-potential at physiological conditions because of negatively charged functional groups associated with various components of the cell membrane (11). The degree of such charges can be measured with zeta-potential (ζ) to estimate surface potential charge and determine the energy required to move cells toward a surface (12). Stage two is marked by the EPS matrix formation. During this step, antimicrobial resistance is observed from the inability of a drug to penetrate the biofilm (13–15). Additionally, the third stage of development shows greater formation of microcolonies containing persister cells as the biofilm surface area increases (16, 17). Matured, late-stage biofilm formation is accompanied by a significant ζ-potential change when compared to their planktonic counterparts (18, 19). The fourth stage encompasses dispersal, allowing the biofilm to release planktonic bacteria and restart the growth cycle (20).

Among antibiotics chosen for treatment of *Staphylococcus* spp., vancomycin and daptomycin are considered the standard of care and recommended treatment options for MRSA (10). However, other broad-spectrum antibiotics, such as levofloxacin, may be used as empiric therapy and can impact biofilm growth (21, 22). Due to the complexity of the biofilm matrix, antibiotics have varying degrees of treatment success, as biofilms can reduce antibiotic susceptibility up to 1,000-fold the minimum inhibitory concentration (MIC) as demonstrated in our lab and others (23–25). Recently, it was noted that inappropriate antibiotic treatment can be counterproductive for *S. aureus* biofilm-associated infections, as it promotes quorum cheating and increases biofilm development (12). Biofilm promotion in antibiotic presence has additionally been noted by others as occurring in a dose-dependent manner and resulting in a more robust biofilm with enhanced adhesion ability (26). Overall, the dynamics of biofilm treatment with antibiotics and resulting resistance requires further investigation. To further investigate, we aimed to define *in vitro* time ranges for MRSA biofilm stages, and, through zeta-potential measurements, provide insight into the development of the stages. We then aimed to elucidate the impact of antibiotic treatment through multiple biofilm susceptibility assays on late-stage biofilms to determine the efficacy of increasing antibiotic concentrations.

## MATERIALS AND METHODS

### Bacterial isolates

Staphylococcal isolates (*n* = 115) exhibiting various biofilm formation capabilities, including 23 unique spa types, were selected from a larger cohort identified in our previous work (Table S1) (11). Isolates were collected from multiple sources at the Providence Veterans Affairs Medical Center in Rhode Island over a 10-year period. Well-documented biofilm controls, wild-type, strong biofilm-forming *Staphylococcus epidermidis* (ATCC 35984), its isogenic accumulation-negative mutant known as M7, and strong biofilm-forming methicillin-susceptible *S. aureus* (ATCC 35556) were selected to serve as standard comparators for growth conditions and reproducibility (11, 13). For ζ-potential and biofilm susceptibility assays, eight unique spa-type MRSA isolates were selected from our collection, and the abovementioned controls (five strong biofilms, five weak biofilms, one accumulation-negative) were used for further testing (Table 1).

**TABLE 1:** Isolates selected for further testing in ζ-potential and biofilm antibiotic susceptibility assays

Isolate	Species	Spa type	Biofilm	OD_570_	MICs (μg/mL)		
					Vancomycin	Daptomycin	Levofloxacin
M7	MRSE	-^*a*^	Weak	0.3	1	0.5	0.125
4620	MRSA	t064	Weak	1.15	1.5	0.5	16
4320	MRSA	t008	Weak	0.88	1	0.5	0.25
40	MRSA	t2666	Weak	0.69	1.5	0.5	32
78	MRSA	t1904	Weak	0.63	2	0.5	32
17	MRSA	t067	Strong	3.28	1.5	0.5	32
884	MRSA	t1340	Strong	3.67	1	0.25	512
398	MRSA	t062	Strong	3.61	1	0.25	32
4413	MRSA	t002	Strong	3.52	1.5	0.5	32
ATCC 35984	MRSE	-	Strong	3.4	2	0.5	0.125
ATCC 35556	MSSA	-	Strong	3.07	1	0.25	16

^*a*^ -, information not known.

### Antimicrobial agents

Antibiotics used were vancomycin (NDC 0409-6533-11; lot: A000038663), levofloxacin purchased from Sigma-Aldrich (CA #28266-10G-F; lot: 0000198729), and daptomycin (NDC 70594-034-01; lots: 3153451, KZ201). Maximum antibiotic concentrations tested were 1,024 μg/mL due to solubility limits in cation-adjusted Mueller–Hinton broth (CA-MHB).

### Susceptibility testing

CA-MHB (Difco Laboratories, Sparks, MD) was used and supplemented with 12.5 mg/L magnesium and 25 or 50 mg/L calcium. Higher concentrations of calcium were utilized for daptomycin activity per Clinical and Laboratory Standards Institute (CLSI) (14). MICs were determined in duplicate in accordance with the CLSI criteria (14, 15).

### Biofilm quantification

Biofilm production was determined using a modified Christensen et al. method as previously described by our group (16–18). Isolates were grown from initial culture stocks stored at −80°C and streaked on tryptic soy agar. Following an incubation period of 18–24 h, a CLSI standard inoculum of 5–6 log_10_ CFU/mL was made, representative of a typical infectious bioburden, in tryptic soy broth with added 25 mg/L calcium and 12.5 mg/L magnesium and supplemented to 1.25% dextrose (STSB) in 96-well polystyrene tissue culture plates (TC-Treated Corning #3596) (19). Each plate was incubated for an assigned duration (2, 4, 6, 8, 16, and 24 h) at 35°C and 50 rpm on an orbital shaker. Planktonic bacteria were removed by inverting plates and rinsing three times with double-distilled sterile water (ddH_2_O) using an electronic pipette (Eppendorf Xplorer) at the lowest setting. Biofilm was quantified in at least eight wells. Uninoculated fresh media were used as a negative control and subtracted from the optical density (OD_570_) results prior to statistical analysis to remove background interference. Plates were inverted after every rinse to remove ddH_2_O and inspected to ensure that there was no remaining liquid before being fixed by air drying overnight in a biosafety cabinet. The fixed biofilms were stained with 0.1% crystal violet for 15 min and rinsed three times with ddH_2_O to remove excess stain before resolubilizing the bound crystal violet with 33% (v/v) glacial acetic acid. Stained biofilms were read at OD_570_ nm using a BioTek spectrophotometer (model ELX800; BioTek, Winooski, VT).

### Biofilm stage definitions

The first stage of the biofilm begins immediately at the 0 h time point, with the starting inoculum representing planktonic cells of a typical infectious bioburden. The identification of the subsequent individual stages of biofilm development will occur at time points after hour 0 to identify significant differences between them.

### ζ-Potential

To further analyze the different properties of biofilms with respect to their stage, we also evaluated electrostatic charges by measuring ζ-potential. Using the above-described biofilm assay, the selected samples of 11 staphylococcal isolates were grown in tissue culture-treated plates, all with the same STSB media to form first- and fourth-stage biofilms. The ζ-potential measurements were conducted in the absence of antibiotics to provide additional insight into biofilm growth characteristics. Bacterial ζ-potential of planktonic cells (0 h) was also determined and compared to early- and late-stage biofilm time points following resuspension. Measuring ζ-potentials was done using a Malvern Zetasizer Nano ZS (Malvern Instruments). ζ-Potentials were calculated from the electrophoretic mobility by Smoluchowski’s equation at 25°C, with five repeats per sample (20, 21). The dielectric constant of the dispersant was set at 78.54. Viscosity was set at 0.6864 cP, and the refractive index was set at 1.333 (22).

### Residual biofilm and viability assays

The effects of three antibiotics (vancomycin, levofloxacin, and daptomycin) with differing mechanisms of action on preformed fourth-stage (24 h) biofilms were determined using a previously described method for measuring viability and residual biomass after antibiotic treatment that uses resazurin and crystal violet staining techniques (23). Resazurin changes color to fluorescent resorufin, which indicates cell health and allows for quantitative measurements of viability within the biofilm (24). Biofilms were grown as described above for 24 h before adding STSB containing serially diluted antibiotics in quadruplicate for 24 h. After treatment, plates were inverted to remove antibiotics, and resazurin was added as previously described. Fluorescence of viable cells was read on a Cytation 5 plate reader at *λ* ex = 560 nm; *λ* em = 590 nm (23). Resazurin was removed by inverting plates, and the remaining biofilm mass was stained with crystal violet before rinsing and measuring residual biofilm (OD_570_) as stated above in the biofilm quantification methods. Biofilm viability was calculated using the previously described formula (image below) and two cutoff values to determine the fluorometric-based viability minimum biofilm eradication concentration (MBEC) for 50 and 75% nonviable cells:


$$cell\;viability\;(\%)=\frac{\left(\text{ corresponding well signal })-(\text{ media control well signal }\right)}{\left(\text{ positive control well signal })-(\text{ media control well signal }\right)}\times 100.$$

### Statistical analysis

An analysis of variance (ANOVA) with post-hoc Tukey’s test was conducted to compare the overall significance between the various time points to classify the different stages of biofilm growth and determine the significance of antibiotic treatment on biofilms. All statistical tests were completed using SPSS software (IBM SPSS Statistics 28.0.1.1) with an *α* value of 0.05 required for significance. Our groups’ previously defined cutoffs will be used to categorize biofilm strengths; weak biofilm formers will have a resulting crystal violet optical density (OD) < 1.0, while strong formers will have an OD > 2.0, as shown in Table 1 (11).

## RESULTS

### Biofilm stage definitions

**Fig 1:**
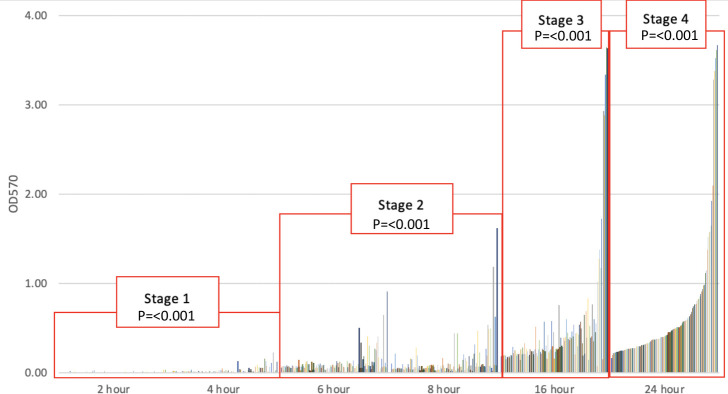
One hundred fifteen MRSA isolate biomass at each time point, with labeled squares showing the different significant stages.

**Fig 2:**
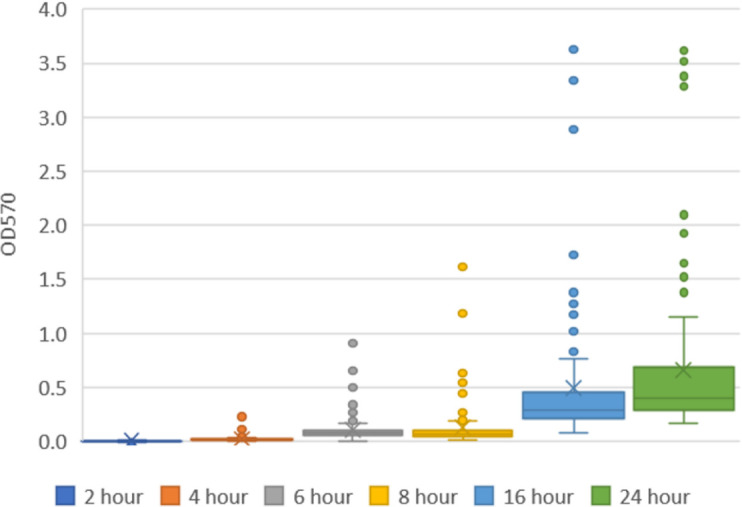
Box plot representation of the 115 MRSA isolate biomass (OD_570_) at each time point. *X* marks the mean, while the line in the box is the median.

Four stages of biofilm development, seen in Fig. 1 and 2, were considered significantly different from each other, and the summary of the statistical analysis results can be found in Table 2. Stage one beginning immediately at the 0 h time point describes the initial attachment that would occur in the presence of an infectious bioburden. This stage spanned the first 6 h with no significant difference in biofilm density detected between the 2 and 4 h marks. Stage two was then identified at 6 h, being significantly different from the 2 (*P* < 0.001) and 4 h (*P* < 0.001) time points. The second stage extended through 16 h, as the 6 and 8 h marks did not significantly differ from each other (*P* > 0.99). The next statistically significant time point identified was at 16 h, denoting the third stage beginning and extending to 24 h. The fourth and final significant stage representing fully matured biofilms began at 24 h, as we observed differing OD readings when compared to 16 h biofilms (*P* < 0.001).

**TABLE 2:** The difference between the different time points was analyzed using an ANOVA model with a Tukey’s post-hoc test^*a*^

Time comparison	Mean difference (95% confidence interval)	*P* value
2–4 h	−0.031 (−0.090, 0.028)	*P* = 0.714
2–6 h	−0.143 (−0.202, –0.084)	*P* < 0.001
4–6 h	−0.112 (−0.171, –0.053)	*P* < 0.001
6–8 h	−0.008 (−0.067, 0.051)	*P* = 1.00
6–16 h	−0.482 (−0.541, –0.423)	*P* < 0.001
8–16 h	−0.473 (−0.532, –0.415)	*P* < 0.001
16 h–24 h	−0.204 (−0.263, –0.145)	*P* < 0.001

^*a*^ The significant *P* value (*P* < 0.05) between measured time points highlights significant differences in growth to define the stages of biofilm development.

### ζ-Potential

After identifying the biofilm stages, we sought to evaluate the electrostatic properties and differences of the bacterial membranes between strong and weak biofilm producers at various stages using mean ζ-potential. For our selected clinical isolates, there was a greater mean difference in ζ-potential charge (mV) observed between planktonic (−4.2 mV) and fourth-stage biofilms (−6.9 mV) for strong biofilm formers (*P* < 0.05) in comparison to the average difference in ζ-potential for weak biofilm formers at planktonic and fourth stages. Conversely, the greatest mean difference averaging the ζ-potential of all clinical MRSA isolates (*P* < 0.05) observed with weak biofilm formers was between planktonic (−6.6 mV) and late first-stage biofilms (−9.2 mV). At all time points, weak biofilm forming isolate mean ζ-potentials were significantly more negative than strong biofilm formers (*P* = ≤ 0.04), as shown in Table 3. The percent change of ζ-potential for strong biofilm producers represents a continuous trend of these isolates becoming more negative as the biofilm stages progress. This trend was interestingly not seen in weak biofilm producers; instead, the fourth-stage biofilms were more positively charged than their late first-stage counterparts. The mean ζ-potential for all mature, fourth-stage biofilms had a more negative ζ-potential than planktonic bacteria (*P* < 0.05).

**TABLE 3:** Mean ζ-potential and percent change over time for high and low biofilm-forming isolates^*a*^

Isolate (spa type)	Biofilm type	Mean ζ-potential (mV)			*Staphylococcus* ζ-potential percent change	
		Planktonic	4 h	24 h	0 to 24 h	4 to 24 h
M7 (ATCC35984 mutant)	Weak	−5.8	−5.1	−5.6	87%	116%
L40	Weak	−8.0	−9.6	−8.5	106%	88%
L78	Weak	−6.8	−9.6	−7.7	113%	79%
L4320	Weak	−4.7	−7.9	−7.4	159%	94%
L4620	Weak	−7.1	−9.7	−7.7	109%	80%
L17	Strong	−4.3	−7.4	−8.5	199%	116%
L398	Strong	−4.6	−4.5	−5.0	109%	112%
L884	Strong	−4.5	−4.9	−8.6	192%	176%
L4413	Strong	−3.5	−4.2	−5.6	161%	133%
ATCC 35984	Strong	−4.5	−4.2	−6.9	153%	165%
ATCC 35556	Strong	−3.6	−4.2	−6.2	172%	148%

^*a*^ Gray highlight indicates a shift to a more positive average charge over time.

### Biofilm viability assay

After establishing Staphylococcal biofilm stages, we conducted cellular viability assays on fourth-stage biofilms in the presence of antibiotics as a surrogate measure for clinically relevant biofilms seen in patients. The vancomycin concentrations tested did not reach either the 50 or 75% viable cell reduction cutoff for weak biofilm-forming isolates. For strong biofilm-forming isolates, vancomycin treatment resulted in at least 50% reduction in viable cells, except for L884. Concentrations that produced this viability reduction ranged from 64 to 1024 μg/mL (representing 32 to 1,024 times the isolate vancomycin MIC) and are specified in Table 4. Levofloxacin was only able to achieve 50% nonviable cells in three isolates, one weak biofilm former and the two strong biofilm controls at concentrations of 8 to 1,024 μg/mL (representing 64 to 4,096 times the levofloxacin MIC). For daptomycin, all isolates achieved at least 50% nonviable cells with concentrations ranging from 8 to 128 µg/mL (representing 16 to 256 times the isolate daptomycin MIC). Unlike the two other antibiotics tested, daptomycin treatment additionally reached the 75% nonviable cutoff for all isolates at concentrations of 32 to 256 μg/mL (respectively reflecting 64 to 512 times the isolate daptomycin MIC).

**TABLE 4:** Fluorometric-based assay minimal biofilm eradication concentrations (MBEC, μg/mL) were categorized by reaching about 50 and 75% of the total nonviable cells and maintaining that percentage or lower as antibiotic concentrations increase

Isolate	Biofilm	Vancomycin		Daptomycin		Levofloxacin	
		MBEC 50	MBEC 75	MBEC 50	MBEC 75	MBEC 50	MBEC 75
M7	Weak	>1,024	>1,024	128	128	32	>1,024
4620	Weak	>1,024	>1,024	128	256	>1,024	>1,024
4320	Weak	>1,024	>1,024	128	128	>1,024	>1,024
40	Weak	>1,024	>1,024	8	32	>1,024	>1,024
78	Weak	>1,024	>1,024	8	64	>1,024	>1,024
17	Strong	512	>1,024	64	256	>1,024	>1,024
884	Strong	>1,024	>1,024	32	128	>1,024	>1,024
398	Strong	1,024	>1,024	64	128	>1,024	>1,024
4413	Strong	1,024	>1,024	32	64	>1,024	>1,024
ATCC 35984	Strong	64	>1,024	8	64	2	>1,024
ATCC 35556	Strong	128	>1,024	64	128	1,024	>1,024

### Residual biofilm assay

**Fig 3:**
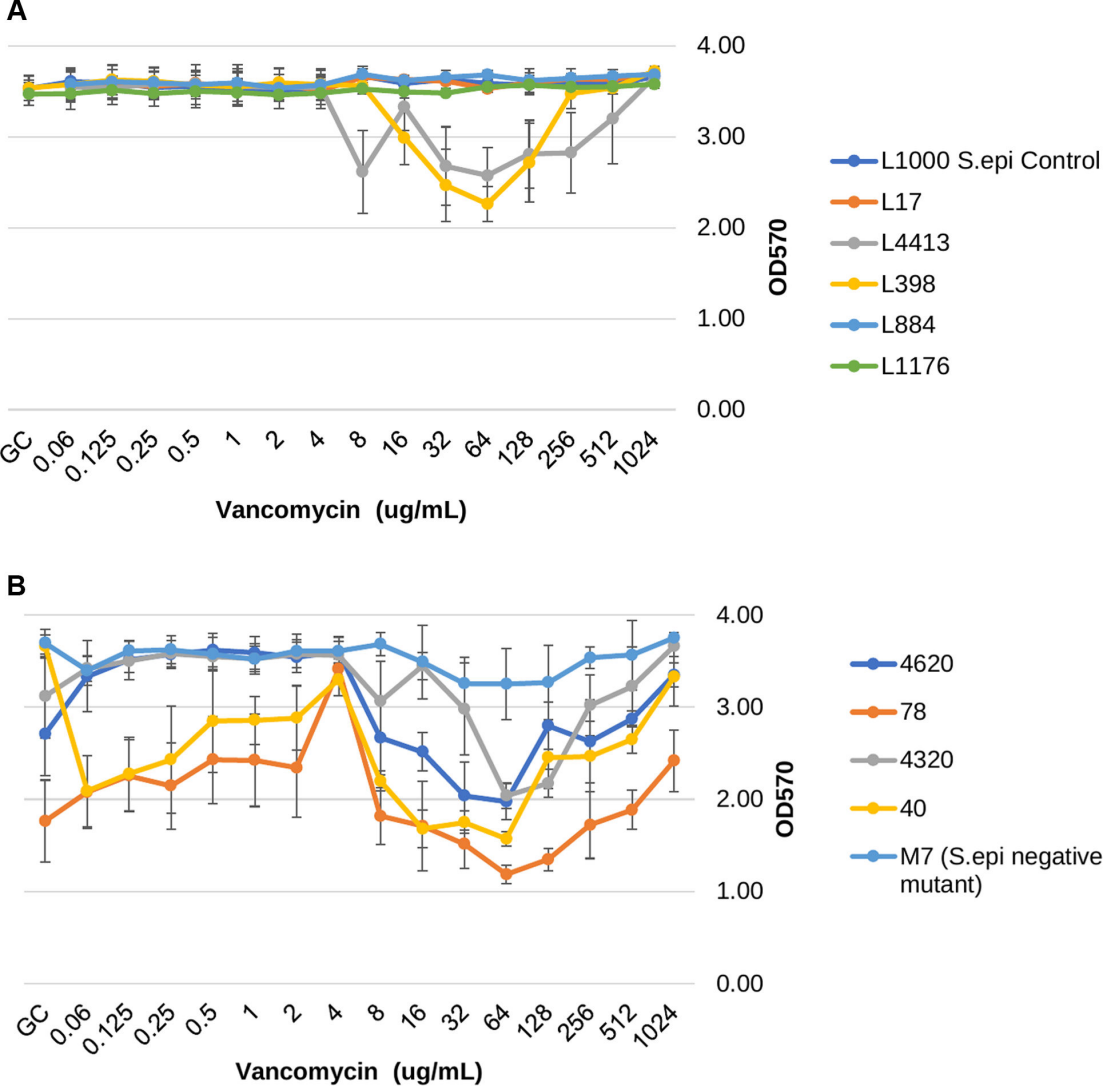
(A) Strong biofilm formers’ remaining biofilm/biomass (OD_570_) after 24 h of growth, followed by 24 h of antibiotic treatment of vancomycin at various concentrations . (**B**) Weak biofilm formers’ remaining biofilm/biomass (OD_570_) after 24 h of growth, followed by 24 h of antibiotic treatment of vancomycin at various concentrations.

**Fig 4:**
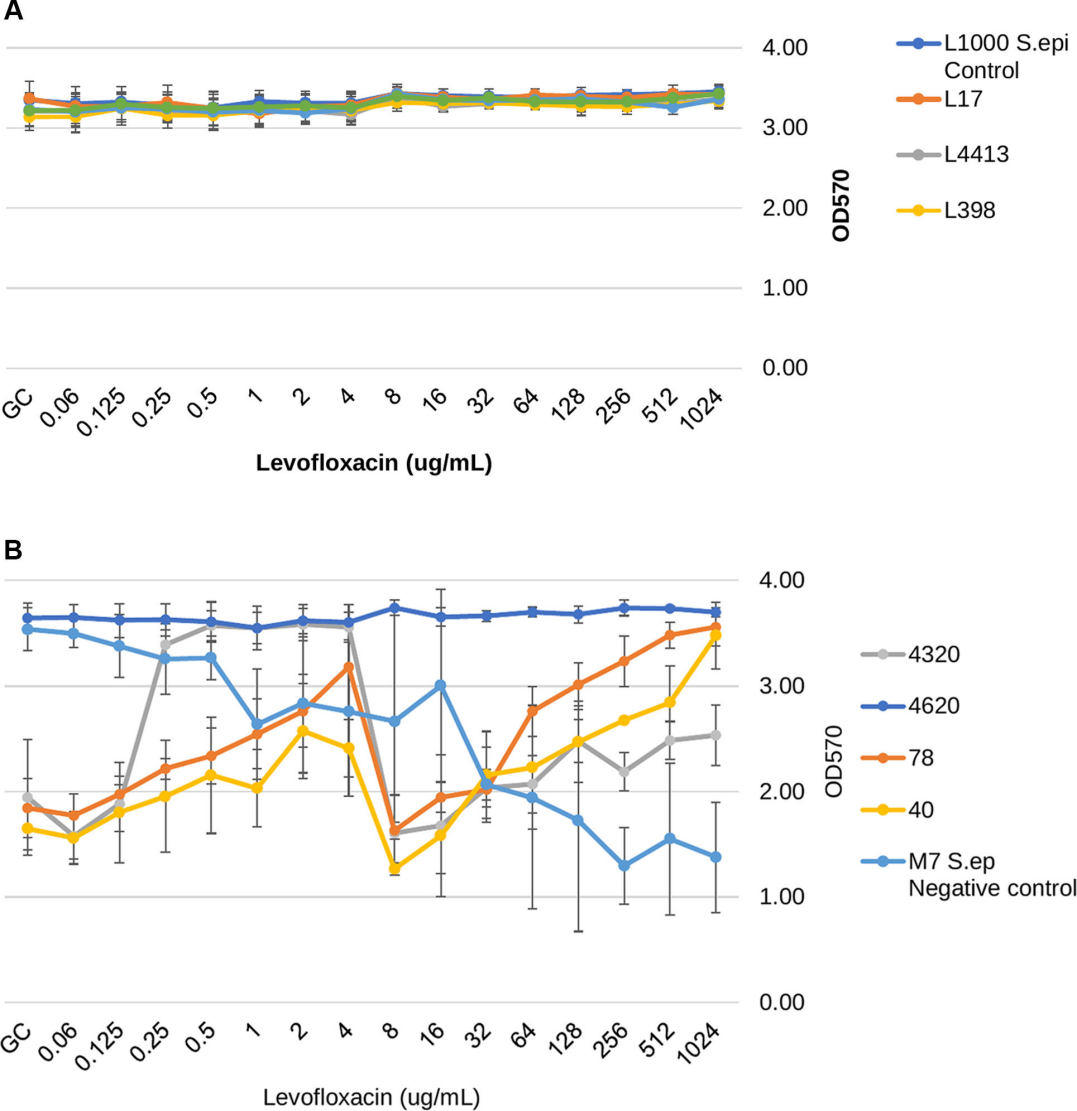
(A) Strong biofilm formers’ remaining biofilm/biomass (OD_570_) after 24 h of growth, followed by 24 h of antibiotic treatment of levofloxacin at various concentrations. (**B**) Weak biofilm formers’ remaining biofilm/biomass (OD_570_) after 24 h of growth, followed by 24 h of antibiotic treatment of levofloxacin at various concentrations.

**Fig 5:**
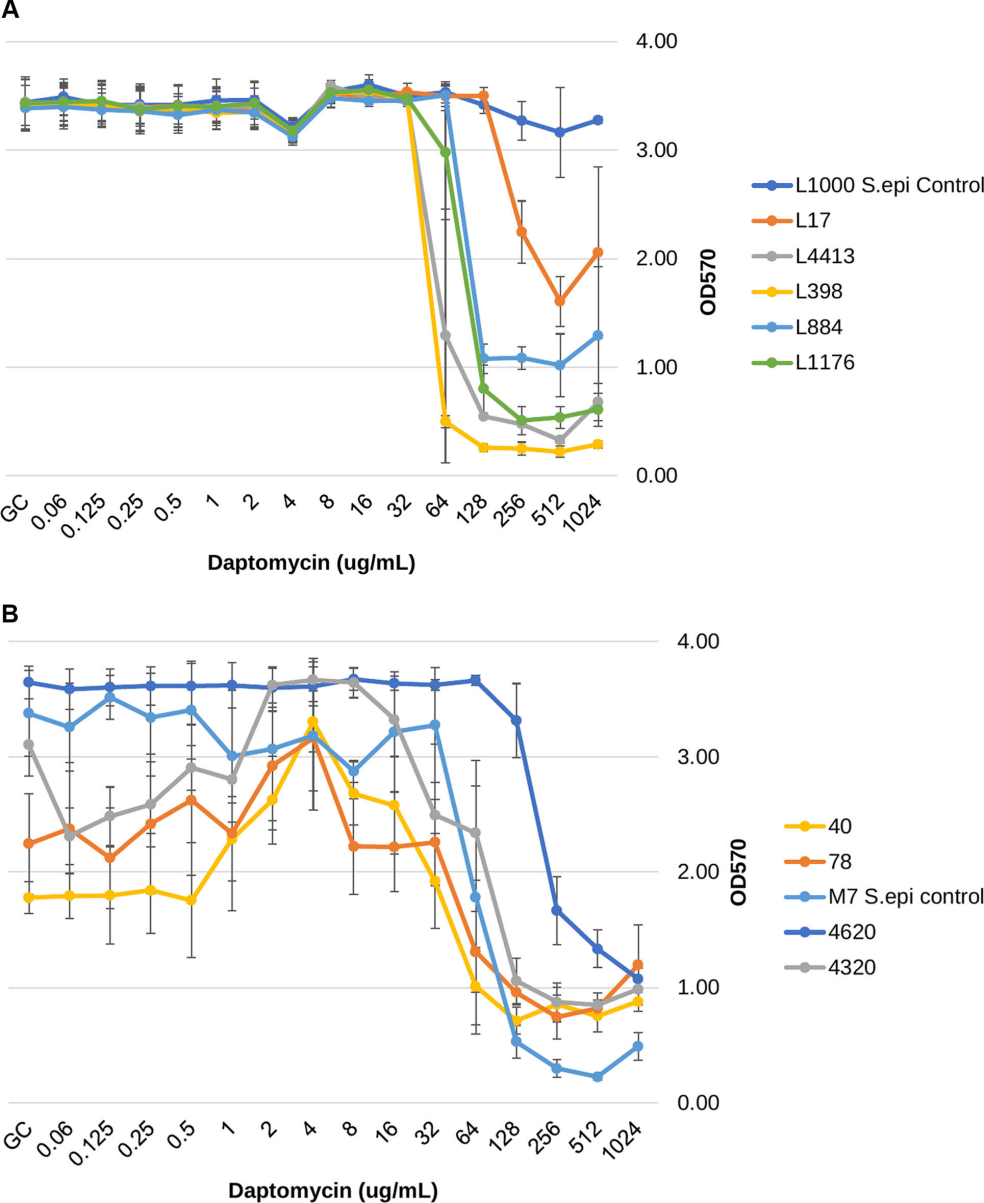
(A) Strong biofilm formers’ remaining biofilm/biomass (OD_570_) after 24 h of growth, followed by 24 h of antibiotic treatment of daptomycin at various concentrations. (B) Weak biofilm formers’ remaining biofilm/biomass (OD_570_) after 24 h of growth, followed by 24 h of antibiotic treatment of daptomycin at various concentrations.

The residual biomass (OD_570_) of biofilms following antibiotic treatment of stage-four biofilms was also evaluated at escalating antibiotic concentrations and compared to the growth control. For the vancomycin-treated, biofilm-embedded isolates, there was a significant reduction in residual biomass for five of the isolates (three weak biofilm producers: L40, L4320, and L4620; and two strong biofilm producers: L398 and L4413), as shown in Fig. 3A and B. Despite producing a significant reduction in nearly half of the tested isolates at concentrations ranging from 8 to 256 µg/mL, there was a subsequently observed increase in OD for all five isolates at higher vancomycin concentrations, reflecting increased biomass or biofilm adherence. Interestingly, for two of the weak biofilm-forming isolates (L78 and L4620), there was conversely a significant increase in residual biomass compared to growth control. All levofloxacin concentrations used did not produce any significant difference in biomass for any strong biofilm-forming isolates, represented in Fig. 4A. The majority of weak biofilm-forming isolates exposed to levofloxacin resulted in a significantly higher residual biomass than their respective growth controls and is shown in Fig. 4B. Daptomycin treatment resulted in a significant reduction in biomass for all MRSA isolates (Fig. 5A and B). Unlike the vancomycin-associated biomass reductions, there was increased adherence solely in the weak biofilm-forming isolate L78 with 1,024 µg/mL for daptomycin.

## DISCUSSION

The development of biofilm from various organisms and environments has been described as occurring in three, four, and five different stages (26–37). Of which, two studies have been conducted in real-time models to specifically evaluate *S. aureus* stages of biofilm (30, 36). First, Moormeier et al. used 10 different *S. aureus* strains with the BioFlux1000 microfluidic flow-cell device system and proposed five stages of development: (i) attachment, (ii) multiplication, (iii) exodus, (iv) maturation, and (v) dispersal (36). Multiplication and exodus, an early dispersal stage, had not been previously discovered or discussed prior to this study (36). Second, Salazar et al. used quartz crystal microbalance-based sensors with *S. aureus* and proposed three observable stages: (i) initial stabilization, (ii) biofilm formation and growth, and (iii) maturation (30). Our study evaluated a cost-effective and readily used *in vitro* microtiter plate biofilm assay method and found a statistical significance between different biofilm stage time points during MRSA biofilm development (Fig. 1). This will enable other researchers to use appropriate time points when running staphylococcal species in the easier biofilm 96-well assay method building upon the foundation of *in vitro* biofilm methods first described by Stepanović et al. (17).

Beginning with the initial attachment of planktonic cells, we consider this first stage to immediately begin at the 0 h time point represented as 5–6 log10 CFU/mL, where attachment occurs following an infectious bioburden of planktonic bacteria and extends through the first 6 h based on the results found in Table 2. Similarly, Moormeier et al. identified rapid progression from his defined stage one, attachment, to stage two, multiplication (36). During the second stage, the biofilm stabilizes from reversible attachment to secure adhesion as the extracellular polymeric substance matrix begins to form, keeping consistent with our definition of stage two, and then followed by the beginning of microcolony formation after proliferation (3, 38). Our results show that stage two is seen at the 6 h time point and continues through 16 h. Salazar et al. also identified significant changes occurring in the first 8 h, with initial stabilization occurring 2 to 8 h and 2 to approximately 5 h (30). Moormeier et al. identified a new stage referred to as exodus having occurred at this same time period from 6 to 11 h and being responsible for an early dispersal phase preceding maturation (36, 38). We cannot identify such changes due to the limitations of using a static model. We then identified a third stage, maturation, at 16 h, which is in accordance with the times described in previous studies. Similarly, Moormeier et al. described maturation occurring from 11 to 18 h, and Salazar et al. identified their own maturation stage to occur within 14–17 h (30). Our last significantly different time point was found to begin at 24 h, ending the biofilm cycle and would likely next be followed by dispersal. At this point, there is believed to be a release of partial or entire biofilm due to nutrient depletion, location inhibition, or survival competition (26). Moormeier et al. also identified the last stage of development as dispersal (36, 38). Salazar et al. failed to identify dispersal; however, when evaluating their results after 24 h, the biofilm growth shows a plateau or a slight decrease from 24 to 48 h, possibly signifying no additional stages and highlighting mature biofilm as dispersal occurs (30).

Prior studies have shown that biofilm formation impacts cellular ζ-potential, exhibiting a higher electronegativity than planktonic cells (39). However, these do not include the comparison of weak and strong biofilm-forming bacteria at different stages of biofilm maturation (40, 41). Staphylococcal biofilms generally possess a negative charge due to electrostatic interactions that arise from the outer bacterial surface, resulting from lipoteichoic acid and extracellular polymer substances. Once establishing biofilm stages, we then compared early- and late-stage biofilm ζ-potential measurements and showed that weak biofilm formers begin surface attachment with higher negative electrostatic charge, while strong biofilm formers begin attachment with a less negative charge before slowly increasing the negative electrostatic charge over 24 h. A higher ζ-potential means more energy is needed for attachment, while a lower repulsion allows bacteria aggregation due to lack of repulsive forces (42). The less negative charge of staphylococcal isolates may be able to predict strong biofilm forming capability.

In order to have a more complete understanding of staphylococcal biofilm optimal antibiotic inhibitory concentrations, we additionally aimed to determine the efficacy of increasing antibiotic concentrations of stage-four biofilms by assessing both. Vancomycin was unable to reduce viability beyond the 50% cutoff for the MRSA isolates and failed to have any appreciable reduction of viability for any weak biofilm isolates. Levofloxacin was largely ineffective at reducing MRSA viability, with only one isolates' viability decreasing by 50%. Viability results showed daptomycin as the more effective antibiotic at reducing viability for both strong and weak biofilm-forming MRSA isolates, with treatment resulting in a ≥75% reduction in viability at concentrations 16 to 512 times the MIC for all isolates. Both levofloxacin and daptomycin had quantifiable MBEC in at least one weak biofilm-forming organism, unlike vancomycin. The lowest MRSA MBEC with either daptomycin or levofloxacin treatment was seen when treating weak biofilm-forming isolates. This is consistent with the understanding that biofilm production confers antimicrobial resistance. This phenomenon being observed with levofloxacin and daptomycin may reflect the pharmacokinetics of these agents, which display more rapid bactericidal activity against planktonic *S. aureus* than vancomycin. These data also coincide with our previous publication showing daptomycin as the more effective mature biofilm treatment option in S. *epidermidis*.

For stage-four biofilm treatment antibiotic assays, in the presence of vancomycin, results were inconsistent and isolate-dependent. Approximately half of isolates saw no change in residual biofilm (OD_570_) in the presence of vancomycin, and, for those with a significant decrease in biofilm following vancomycin treatment, there was an immediate increased biofilm biomass seen at escalating vancomycin concentrations. Additionally, two weak biofilm formers (L78 and L4620) saw significantly stronger biofilm biomass (*P* < 0.05) at higher vancomycin concentrations (Fig. 3B). Interestingly, the intermediate vancomycin concentrations were associated with the greatest reduction in OD for all but two MRSA isolates, suggesting there may be an optimal antibiotic concentration window that should be targeted for maximum biomass reduction. Further studies are warranted to further elucidate this effect.

In the presence of levofloxacin, all strong biofilm formers’ residual biofilm was unchanged compared to growth control (Fig. 4A). For weak biofilm formers, in the presence of levofloxacin, three isolates resulted in a significantly stronger residual biofilm (*P* = <0.05), while one isolate remained unchanged compared to growth control. The accumulation of negative biofilm control (M7) resulted in a significantly lower biomass at max concentrations (*P* = <0.05) (Fig. 4B). After treatment with daptomycin, all strong and weak biofilm-forming isolates had their residual biomass significantly decrease (*P* < 0.05) (Fig. 5A and B). However, one weak biofilm isolate (L78) showed some increased biofilm adherence, though not significant, at the highest concentration of 1,024 μg/mL. The weak biofilm-forming isolate L40 at lower concentrations showed a significant increase in biomass compared to growth control (*P* < 0.05) before the significant biomass decrease was seen at 128 to 1,024 μg/mL.

Our residual biomass findings unexpectedly showed that, after an initial residual biofilm (OD_570_) decrease in response to specific concentrations of antibiotics, biofilm biomass increased at higher antibiotic concentrations. Higher antibiotic concentrations may have been anecdotally thought to bypass biofilm resistance and eliminate higher-burden infections; however, these results illuminate that there instead may be a treatment concentration window to achieve optimal kill. Here, we denote the progression from planktonic organisms to preformed staphylococcal biofilms, and then to stage-four mature biofilms accompanied by the resulting stress-response data caused by increasing antibiotic treatment.

We identify several limitations within our study. First, the microtiter plate biofilm assay does not allow for real-time continuous results, but the similarity between the previous studies’ results allows us to extend our stage classifications. Additionally, we utilized *S. epidermidis* isolates as well-established controls for standard *in vitro* biofilm growth conditions; however, it should be noted that biofilm formation of *S. aures* and *S. epidermidis* is not ubiquitous (43). Next, we acknowledge that using 96-well microtiter plates may lead to an exhaustion of available nutrients or biological space and, therefore, limits biofilm. When evaluating results over 24 h in an open model, the results plateau between the 16 and 24 h time points, which leads us to believe that our closed model reached a fully mature and stable biofilm that has or was about to begin dispersal. Last, although a strength of our study is our sample of 115 MRSA isolates for stage determination, we had a majority of weak biofilm-forming isolates. Levofloxacin MIC ranges of tested isolates were large (0.125–512 μg/mL), while vancomycin and daptomycin MICs were similar. Future research is needed to determine the clinical application and effect of biofilm-forming strengths in relation to antibiotic efficacy and assess if bacterial membrane electronegativity changes impact biofilm growth.

### Conclusion

Using 115 unique MRSA isolates, we utilized a cost-effective readily reproducible assay to identify four stages of biofilm growth at the following time points: stage one is from 0 to 6 h; stage two is from 6 to 16 h; stage three is from 16 to 24 h; and stage four is from 24 h onward. All isolates exhibited growth at their own individual rates, but we were still able to see a statistically significant difference across multiple time points. We then observed a trend of weak biofilm formers beginning surface attachment with a higher negative electrostatic charge and strong biofilm formers beginning attachment with lower negative charges before slowly increasing the negative electrostatic charge over 24 h. Optimal antibiotic eradication concentrations for stage-four mature biofilms were explored, and daptomycin was the only antibiotic that resulted in all tested isolates having ≥75% reduction in viability and residual biofilm (OD_570_) significantly reduced when compared to vancomycin and levofloxacin. Further work is needed to mechanistically understand the increased biofilm biomass caused by higher antibiotic concentrations seen in stage-four antibiotic-treated biofilms.

## SUPPLEMENTAL MATERIAL

Table S1 spectrum.02915-24-s0001.pdf Selected MRSA isolates from various sources with unique spa types and different levels of biofilm production.

ASM does not own the copyrights to Supplemental Material that may be linked to, or accessed through, an article. The authors have granted ASM a non-exclusive, world-wide license to publish the Supplemental Material files. Please contact the corresponding author directly for reuse.
