# Supplementary material for: Staphylococcus biofilm dynamics and antibiotic resistance: insights into biofilm stages, zeta potential dynamics, and antibiotic susceptibility
Source: Microbiol Spectr. 2025 Mar 26;13(5):e02915-24. doi: 10.1128/spectrum.02915-24 (PMC12054104; doi:10.1128/spectrum.02915-24)
Supplement: Table S1 — Selected MRSA isolates from various sources with unique spa types and different levels of biofilm production. [file spectrum.02915-24-s0001.pdf]

Supplementary TABLES AND FIGURES

ID	Optical Density	Biofilm Production	Source	Spatype[1]	MLSTcc[2]
**17**	3.28	Strong	Nares	t067	5
**22**	0.28	Weak	Blood	t064	8
**28**	0.24	Weak	Blood	t895	5
**29**	0.25	Weak	Blood	t895	5
**30**	0.23	Weak	Blood	t895	5
**31**	0.27	Weak	Blood	t895	5
**32**	1.52	Moderate	Blood	t895	5
**37**	0.43	Weak	Tissue	t002	5
**40**	0.69	Weak	Blood	t2666	5
**53**	0.20	Weak	Blood	t895	5
**57**	0.33	Weak	Blood Catheter	t895	5
**59**	0.40	Weak	Tissue	t002	5
**63**	1.11	Moderate	Tissue	t062	8
**74**	0.36	Weak	Urine	t002	5
**78**	0.64	Weak	Tissue	t1904	45
**82**	0.37	Weak	Blood Catheter	t1094	5
**83**	0.59	Weak	Blood	t1094	5
**91**	0.49	Weak	Tissue	t008	8
**100**	0.57	Weak	Urine	-	-
**103**	0.27	Weak	Urine	t088	5
**108**	0.38	Weak	Urine Catheter	t002	5
**109**	0.22	Weak	Tissue	t895	5
**117**	0.62	Weak	Tissue	t693	1
**120**	0.25	Weak	Blood	t895	5
**123**	0.28	Weak	Urine	t008	8
**125***	0.91	Weak	Urine	t004	45
**131**	1.38	Moderate	Urine Catheter	t088	5
**132**	0.65	Weak	Urine Catheter	t242	5
**146**	0.24	Weak	Urine	t895	5
**159**	0.98	Weak	Urine	t189	1
**170**	0.53	Weak	Tissue	t002	5
**173**	0.47	Weak	Tissue	t018	30
**174**	0.62	Weak	Tissue	t008	8
**183**	0.83	Weak	Tissue	t064	8
**185**	1.58	Moderate	Tissue	t334	8
**198**	0.77	Weak	Blood	t548	5
**199***	0.27	Weak	other	t895	5
**201**	0.33	Weak	Tissue	t002	5

**216***	0.94	Weak	Tissue	t010	5
**218**	0.45	Weak	Blood	t002	5
**219**	0.35	Weak	Urine Catheter	t002	5
**230**	0.41	Weak	Blood	t1094	5
**233**	0.30	Weak	other	t002	5
**237**	0.39	Weak	Tissue	t002	5
**244**	0.33	Weak	Tissue	t895	5
**259**	0.32	Weak	Tissue	t895	5
**260**	0.48	Weak	Urine Catheter	t002	5
**271**	0.55	Weak	Urine	t002	5
**272**	0.99	Weak	Urine	t002	5
**280**	0.51	Weak	-	t002	5
**301***	0.37	Weak	-	-	-
**333**	0.81	Weak	Tissue	t064	8
**334**	0.49	Weak	Urine	t008	8
**374**	0.51	Weak	Tissue	t2032	8
**379**	1.57	Moderate	Tissue	-	-
**398**	3.62	Strong	other	t062	8
**448**	0.31	Weak	Blood Catheter	t002	5
**449**	0.30	Weak	Tissue	t002	5
**456**	1.65	Moderate	Urine	-	-
**468**	0.25	Weak	Blood	t895	5
**469**	0.50	Weak	Urine	t002	5
**500**	0.22	Weak	Urine Catheter	t002	5
**519**	0.73	Weak	Tissue	t1094	5
**535**	0.58	Weak	Blood	t002	5
**575**	0.40	Weak	Tissue	t008	8
**672**	0.46	Weak	Tissue	t008	8
**678**	0.37	Weak	Urine Catheter	t002	5
**781**	0.27	Weak	Urine	t985	5
**818***	0.77	Weak	Blood	t002	5
**834**	0.28	Weak	Blood	t895	5
**840**	0.46	Weak	Blood	t895	5
**846**	0.28	Weak	Tissue	t010	5
**884**	3.66	Strong	Urine Catheter	t1340	5
**909**	0.83	Weak	Tissue	t008	8
**977**	2.10	Strong	Tissue	t002	5
**4056**	0.75	Weak	Urine	t1094	5
**4124**	0.38	Weak	Urine Catheter	t008	8
**4156**	0.32	Weak	Urine	t895	5
**4160**	0.40	Weak	Tissue	t088	5
**4174**	0.52	Weak	Blood	t681	8
**4181**	0.30	Weak	Urine Catheter	t895	5
**4183**	0.42	Weak	Blood	t002	5
**4283**	0.31	Weak	Blood Catheter	t002	5
**4289**	0.27	Weak	Blood	t067	5
**4293**	0.77	Weak	Blood	t008	8
**4297**	0.24	Weak	Urine	t067	4

**4301**	0.38	Weak	Urine	t008	8
**4320***	0.88	Weak	Tissue	t008	8
**4332**	0.27	Weak	Tissue	t008	8
**4354**	0.23	Weak	Blood	t242	5
**4355**	0.40	Weak	Tissue	t008	8
**4356**	0.59	Weak	Urine	t008	8
**4388**	0.29	Weak	Blood	t002	5
**4413**	3.52	Strong	Urine Catheter	t002	5
**4423**	0.51	Weak	Urine Catheter	t002	5
**4456**	0.27	Weak	Tissue	t002	5
**4533**	0.25	Weak	Urine	t002	5
**4612**	0.40	Weak	Blood	t1094	5
**4620**	1.15	Moderate	Blood	t064	8
**4637**	0.35	Weak	Urine Catheter	t002	5
**4654**	0.51	Weak	Urine	t002	5
**4661**	0.26	Weak	Urine	t002	5
**4675**	0.16	Moderate	Blood	t895	5
**4701**	0.38	Weak	Urine	t002	5
**4715**	1.93	Moderate	Nares	t4223	8
**4721**	0.38	Weak	Blood	-	-
**4726**	0.31	Weak	Blood	t008	8
**4768**	0.26	Weak	Urine Catheter	t008	8
**4795***	0.30	Weak	Blood	t008	8
**4823**	0.58	Weak	Urine Catheter	t1094	5
**4849**	0.32	Weak	Blood	t002	5
**4950**	0.85	Weak	Urine Catheter	t002	5
**4951**	0.25	Weak	Urine	t002	5
**4954**	3.38	Strong	Blood	t002	5
**4998**	0.50	Weak	Urine	t002	5

[1]Staphylococcus aureus protein A
[2]Multi locus sequence testing, clonal complex
*Heteroresistant vancomycin-intermediate *Staphylococcus aureus* (hVISA)

Supplementary Table 1. Selected MRSA isolates from various sources with unique Spa types and different levels of biofilm productions.
